# The evolution of activity breaks in the nest cycle of annual eusocial bees: a model of delayed exponential growth

**DOI:** 10.1186/1471-2148-6-45

**Published:** 2006-06-02

**Authors:** Oliver Mitesser, Norbert Weissel, Erhard Strohm, Hans-Joachim Poethke

**Affiliations:** 1 Forschungsstation Fabrikschleichach, Universität Würzburg, Glashüttenstr. 5, D-96181 Rauhenebrach, Germany; 2 Biozentrum (Zoologie III), Universität Würzburg, Am Hubland, D-97074 Würzburg, Germany; 3 Institut für Zoologie, Universität Regensburg, Universitätsstr. 31, 93040 Regensburg, Germany

## Abstract

**Background:**

Social insects show considerable variability not only in social organisation but also in the temporal pattern of nest cycles. In annual eusocial sweat bees, nest cycles typically consist of a sequence of distinct phases of activity (queen or workers collect food, construct, and provision brood cells) and inactivity (nest is closed). Since the flight season is limited to the time of the year with sufficiently high temperatures and resource availability, every break reduces the potential for foraging and, thus, the productivity of a colony. This apparent waste of time has not gained much attention.

**Results:**

We present a model that explains the evolution of activity breaks by assuming differential mortality during active and inactive phases and a limited rate of development of larvae, both reasonable assumptions. The model predicts a systematic temporal structure of breaks at certain times in the season which increase the fitness of a colony. The predicted pattern of these breaks is in excellent accordance with field data on the nest cycle of the halictid *Lasioglossum malachurum*.

**Conclusion:**

Activity breaks are a counter-intuitive outcome of varying mortality rates that maximise the reproductive output of primitively eusocial nests.

## Background

Some of the most important components of life history decisions refer to the optimal timing of accumulation of resources and their allocation to growth and reproduction. At any time during its life an organism has not only to spend resources on the conflicting requirements for maintenance, somatic growth, and reproduction, but has also to decide on how much and when resources like food or building material should be accumulated in order to maximise reproductive output. Up to now, theoretical studies on life history strategies of eusocial insects have mainly focused on the first aspect: optimal resource allocation [1-4]. However, the obvious and ample variability in seasonal activity patterns within and between species of eusocial insects requires investigating the optimal timing of resource accumulation too.

Seasonal activity patterns vary widely among the species of bees and wasps that have been studied as model organisms for the evolution of sociality in insects [5-7]. Many annual *Polistes*, *Vespa*, *Xylocopa *and *Allodape *species show continuous colony activity during the whole season [8-11]. This results in a more or less continuous production of offspring as is assumed in the classical model of colony development by Macevicz and Oster (1976). Model predictions have been tested and were met in field data from *Polistes *and *Vespa *species [1].

However, the nest cycle of most halictids (e.g. in the genera *Lasioglossum *and *Halictus*) is characterized by several discrete broods that are separated by distinct activity breaks [[6,12,13], but see [14] and [15]]. During the solitary founding phase, halictid queens construct nests and supply brood cells with pollen and nectar as provisions for their larvae. After a break of a few weeks, during which the nest is closed and no activity outside the nest can be observed, a first worker brood emerges and starts collecting pollen and nectar to provision the eggs that are usually laid by the queen. Subsequent broods are also separated by breaks during which nests are closed and no outside activity can be observed. Activity breaks can last up to three weeks [[12,16], Weissel et al., submitted]. Usually sexuals emerge in the last brood only, while all other broods consist mainly of workers. There are also species with an intermediate position between continuous growth and discrete broods. In *Bombus *species, for example, the nest cycle is organized in more or less discrete broods but without activity breaks and nest closure [17].

Due to temperature-dependence of their activity and resource availability, ectothermic organisms, like insects, have to adjust their life history to the seasonal conditions in temperate latitudes. Reproduction and growth must be completed within a limited time span and the unfavourable period has to be bridged by diapause. Variability in biotic and abiotic conditions during the reproductive period has been assumed to cause changes in activity patterns on a smaller time scale [18]. Usually bees will forage during the day when visibility is good and temperature is high enough for flying and foraging [19-23]. However, the evolutionary transitions to dim-light foraging have occurred repeatedly in bees, and may be associated with the escape from enemies or competitors [24,25]. The daily activity patterns of the solitary bee *Anthophora plumipes *has been attributed to variation in the thermal environment as well as quality and quantity of floral resources [26]. The pattern of activity breaks in halictids has accordingly been related to patterns of resource availability and photoperiod [14,16,27]. By contrast, Kaitala et al.'s (1990) modelling approach for *L. malachurum *assumes synchronized nest closure in halictid nest aggregation to be due to an increasing threat of nest usurpation by intraspecific parasites, so called floater queens.

Furthermore one could suppose that activity breaks after the emergence of the first workers just appear when worker mortality is rather high and all workers of a brood have died before emergence of the individuals of a second brood. However, it is clear from field observations that the breaks do not occur simply because all workers of a brood have died. Some workers even survive a complete activity break and continue foraging when the nest is reopened [[16], Weissel and Strohm unpublished]. On the contrary, breaks occur even though there are still some workers alive in a nest, showing that there has to be some advantage of interrupting foraging activity.

The well-known colony growth model of Macevicz & Oster (1976) for insect colonies identifies the sequential production of workers first and sexuals just before the ending of the flight season (so called bang-bang reproduction) as the optimal investment strategy to maximize colony fitness. Whereas this model assumes instantaneous occurrence of adult progeny the model that we present accounts for a certain development time of the larvae. The results of our model challenge the assumption that only variation in environmental factors governs the emergence of activity breaks. The model explains the evolution of the observed activity patterns rather by an asymmetric interaction between endogenous and exogenous factors of colony development.

## Results

### A simple model of delayed exponential growth with activity breaks

We use a simple difference equation model to analyse colony development during a season of length *L*. Two main dependent variables describe the state of a colony: the number of workers (*W*_*i*_) at time step *i *and the number of sexuals (*S*_*i*_) at that time. For simplicity we do not distinguish between male and female sexuals [1]. The colony cycle typically starts in spring with nest founding by inseminated hibernated queens. During the founding phase the queen works alone and performs all the foraging tasks that will be taken over by workers after their emergence later in the season [7]. Thus we start with initial condition *W*_1 _= 1 assuming that the founding queen acts like a single worker until the first eggs have developed to adults [16]. The dynamics in the number of nestmates is governed by two mechanisms: mortality and reproduction. Each individual survives from time step *i *to *i *+ 1 with a probability *q*_*i *_(that might vary with time step *i *during the season). Resource allocation in each time step (*i*) is directly proportional to the current worker force (*W*_*i*_). Each worker can provision *c*_*i *_(worker efficiency) eggs (= brood cells) per time step. We assume that the actual egg laying rate of the queen is only limited by the number of eggs that can be successfully provisioned by the workers of the colony [16]. Adults emerge after a development time *T*. Halictid colonies suffer from numerous threats during activity periods (see discussion), so nest and especially brood mortality are rather high [Weissel et al., submitted]. As this parameter is not in the main focus of our analysis and field data are not readily available, we use the same survival probability (*q*_*i*_) for eggs and for adult workers to keep the model simple (Additional numerical calculations have shown that our results differ only quantitatively if we uncouple worker and brood mortality). Additionally we assume that development time (*T*) does not correlate with either season length (*L*), caste or onset of development.

The portion *u*_*i *_of resources spent in time step *i *is allocated to new workers. Consequently, the portion (1 - *u*_*i*_) is invested in sexuals *S*_*i*_. Thus, the number of workers (*W*_*i*+1_) at time step *i*+1 can be calculated as

*W*_*i*+1 _= *q*_*i *_*W*_*i *_+ *u*_*i *_*c*_*i*-*T *_*W*_*i*-*T *_Π^*i*^_*i*-*T *_*q*_*i*_ (1)

In most halictid bees, life span of adult females is much longer than life span of workers [12]. Female sexuals have to hibernate before nest founding in the following year, while workers live only for several weeks. Thus we neglect mortality of sexuals as has been done by Oster and Macevicz (1976) in most of their analyses and thus we get for the number of sexuals (*S*_*i*+1_) at time step *i*+1

*S*_*i*+1 _= *S*_*i *_+ (1 - *u*_*i*_)*c*_*i*-*T*_*W*_*i*-*T*_Π^*i*^_*i*-*T*_*q*_*i *_    (2)

These two equations describe the delayed (by development time *T*) exponential growth of an annual, primitively eusocial bee colony. Fitness of colonies following such nest dynamics can be measured by the final number of sexuals *S*_*L*_. Oster and Wilson (1978) have studied such systems (in time continuous form and without delay) as optimal control problems with control variable *u*_*i *_(allocation in workers) [28]. They found that the (time-dependent) optimal control solution that maximizes *S*_*L *_is switching in *u*_*i *_from 1 to 0 at an optimal point in time (dichotomous bang-bang strategy, *SWT = *switching time). So the optimal temporal pattern of reproduction consists of two distinct phases: exclusive worker production followed by exclusive sexual production. This result also holds for delay systems [2,29]. In our simple model the optimal switching point can be found by a simple argument: Switching should take place when an egg just laid can not mature, eclose and contribute to rearing other individuals anymore. From time *L *- *T *to *L *no eggs should be produced at all, because they would not emerge before the season ends. The last contribution of a worker to sexual production can occur at time *L *- *T *- 1 and thus the last worker egg should be laid at *L *- 2*T *- 1. So we choose



for further analysis.

**Figure 1:**
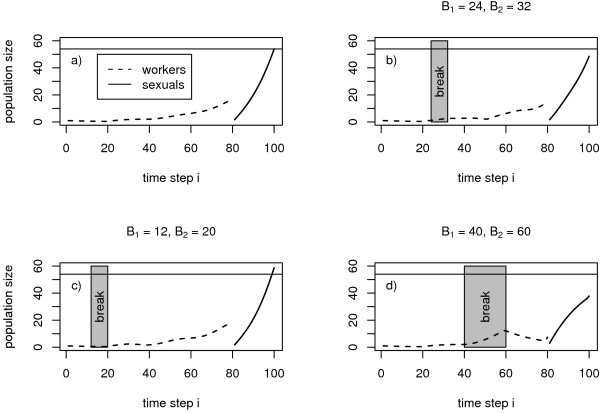
**Development of worker and sexual numbers with and without a break**. Different activity breaks can result in a decrease (b, d) or an increase in the overall production of sexuals (c). Model parameters: worker efficiency *c *= 0.7, off-break survival *q *= 0.95, development time *T *= 20, season length *L *= 100 and within-break survival *Q *= 0.985. The parameters *B*_1 _and *B*_2 _printed above each graph denote the onset and end of the break in question.



We assume constant survival (*q*_*i *_= *q*) and constant worker efficiency (*c*_*i *_= *c*) throughout the whole season. If we ignore the influence of activity breaks on these parameters, we get nest dynamics as shown in figure 1a. To take activity breaks into account, we have to modify both parameters during breaks. Each break starts at time *B*_1 _and ends at *B*_2_. During a break food allocation is impossible (*c *= 0) but survival probability is increased from *q *to *Q *> *q*. Accordingly we formulate

and



Analogously additional activity breaks can be inserted into the nest cycle. However, here we focus on a single break as our main results are not changed by the simultaneous consideration of several breaks. To answer the question of whether there are activity breaks that increase fitness when inserted into the nest cycle we analysed the complete *B*_1_-*B*_2 _parameter space by simple numerical calculations. We interpret *B*_1 _and *B*_2 _as life history parameters of the queen, who decides when to close and reopen her nest [16]. During a single *B*_1_-*B*_2 _space simulation all other parameters were kept constant. Computer simulations are conducted with the programming language R [30].

### Estimating model parameters

We calibrated the model with data from the halictid species *L. malachurum*. In this species a typical season in central Europe lasts for about 100 to 140 days. Since the absolute length of the season did not change our results within this range we choose *L *= 100. The mean life time of *L. malachurum *workers is about 24 days [16]. This results in a survival probability of *q = *0.95 per time step. Development from egg to adult typically lasts *T = *20 time steps [16]. There are no data available about the shelter effect of nest closure, so we studied the effect of within-break survival *Q *in the range from *q *(= 0.95) to 1. Worker efficiency *c *from 0.5 to 1.0 (per time step) results in an output of about 20 to 80 sexuals, similar to typical colony sizes in field observations [31].

### Numerical results

**Figure 2:**
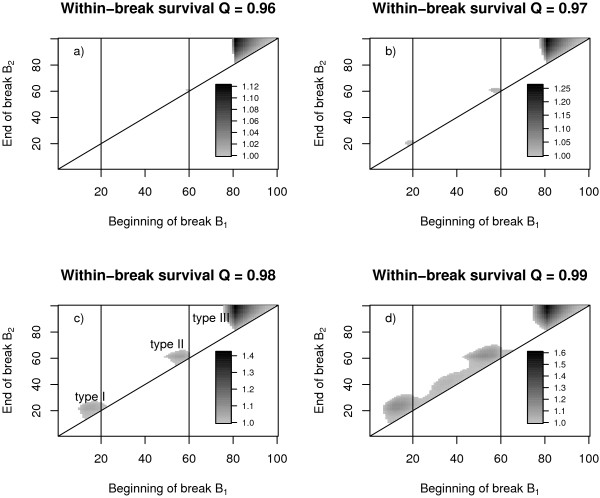
**Relative fitness of single break strategies **(compared to the case without a break) as a function of the time steps at the beginning (*B*_1_) and the end of a break (*B*_2_). Gray areas indicate beginning and end of fitness increasing breaks (shading of areas gives relative fitness of colonies following a respective break strategy). The different figures correspond to scenarios differing in within-break survival *Q*. Increasing within-break survival results in the emergence of two additional areas that represent fitness increasing breaks and which increase in size (b, c, d) with increasing protection during the breaks. For referring to the three prominent areas of fitness increasing breaks in the text we labelled them type I, II and III (c). Model parameters: worker efficiency *c *= 0.5, off-break survival *q *= 0.95, development time *T *= 20, season length *L *= 100.

The relative fitness gain due to activity breaks is shown in figure 2. Gray areas mark *B*_1_-*B*_2 _combinations that result in increased fitness compared to the case without breaks. This corresponds to nesting patterns which yield higher numbers of sexuals at the end of the season (see figure 1b in comparison to 1a and 1c). To facilitate reference to the three prominent areas of fitness increasing breaks we labelled them type I, II and III (see fig. 2c). If activity breaks increase survival probability only slightly (fig. 2a) they will result in a net benefit for colony fitness only at a time where they do not cause any costs. This is the case for breaks which protect the development of the sexuals at the end of the season (type III breaks). Such breaks do not cause any costs because only resources acquired (and allocated to the provisioning of workers or sexuals) before the last development period (of length *T*) will increase the colony's output of sexuals.

However, increased protection during breaks (fig. 2b–2d) causes a second and a third area of beneficial times for breaks (fig. 2b and 2c) (type I and II). The position of these spots remains constant whereas their area increases with increasing break survival probability rate *Q *resulting in an extended area of beneficial break timings.

**Figure 3:**
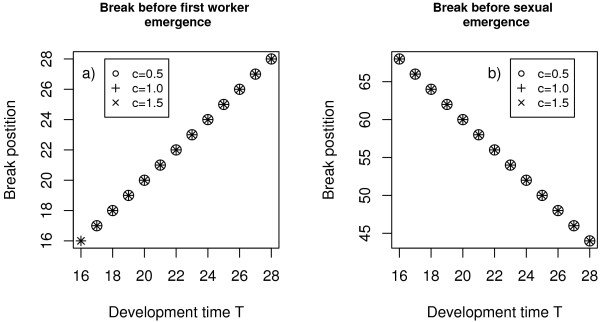
**Temporal position of first (a) and second break (b) as a function of development time and worker efficiency**. Since break duration differs when worker efficiency *c *and development time *T *are changed, optimal break timing was estimated for each parameter combination, when within-break *Q *was just high enough to ensure the emergence of a (very short) fitness increasing break. Model parameters: off-break survival *q *= 0.95, season length *L *= 100. Within-break survival (*Q*) was chosen sufficiently high to ensure the emergence of breaks of minimum duration.

For a more detailed analysis of the temporal position of the type I and II breaks we first calculated the optimal length and position of the trivial break (type III, see above and fig. 2) and based all further analysis on a colony cycle including this optimal type III break. The temporal position of the type I and II breaks was then investigated in relation to development time. In each scenario with fixed model parameters *c *(efficiency), *q *(survival probability) and particular development time *T *we chose the minimal *Q*-value (within break survival) to ensure break emergence of type I and type II respectively. This results in a linear relationship between development time *T *and break position for break types I and II (fig. 3a and 3b). So breaks of type I can be interpreted as breaks just before the emergence of the first workers. And breaks of type II are breaks shortly before the production switch (*SWT*) from workers to sexuals. The results show broad stability over a wide range of the efficiency parameter *c*.

**Figure 4:**
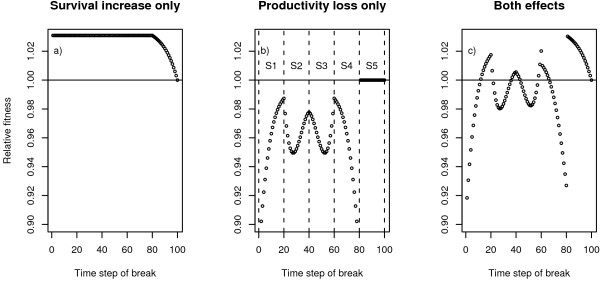
**Influence of the temporal position of short (lasting only for a single time step) activity breaks on relative colony fitness **in different scenarios: a) when productivity is not decreased during breaks (*c*_*i *_= *c*), b) when survival is not increased during breaks (*Q *= *q*) and c) when both mechanisms are kept in the model. Model parameters: worker efficiency *c *= 0.5, off-break survival *q *= 0.97, development time *T *= 20, season length *L *= 100 and within-break survival *Q *= 1.0.

For a deeper analysis of the mechanisms responsible for the position of activity breaks we slightly modified our model and allowed only activity breaks lasting for exactly one time step. We first focused on the effect of mortality and ignored the reduction in worker efficiency during breaks (the loss part in fitness balance). Thus, activity breaks only increase survival but do not reduce resource allocation (fig. 4a). This increases relative fitness (compared to the standard scenario without modification) by a constant factor until the first sexuals emerge. As long as brood is produced the increase in survival (lasting for one time step) operates as a multiplier of the final nest output regardless of the actual time step it happens (see eqn. 1). Thus, an increase in survival of 1% translates directly into a fitness gain of 1% as all sexuals profit from the benefit. As soon as the first sexuals emerge (*T *time steps before the season ends) a break can only protect the development of the remaining brood and consequently the beneficial effect of increased survival declines with each emerging sexual.

Next we reduced efficiency during breaks but kept survival rate (the gain part in fitness balance) constant. This has a more complex effect (fig. 4b). In the last period of the colony cycle (fig. 4b, S5) brood production has ceased and thus, fitness is not affected by late reduction in efficiency. During the prior period (starting at switching time *SWT*, fig. 4b, S4) only eggs for sexuals are laid which contribute to fitness in an additive way. Consequently fitness loss (caused by reduced worker efficiency) is directly proportional to number of workers and as worker number increases during this period, fitness decreases. During the solitary phase of colony development (fig. 4b, S1) sensitivity to productivity loss decreases with time (and relative fitness increases). This effect is not changed at all by different mortality rates (not shown in figure 4b). It is solely caused by the decreasing value of eggs developing into workers. The later a worker egg is produced in the season the less it contributes to overall fitness. At the beginning of the intermediate periods (S2 and S3) the first workers emerge and the oscillating pattern of the relative fitness function is governed by the interaction of both processes acting separately in periods S1 and S4.

**Figure 5:**
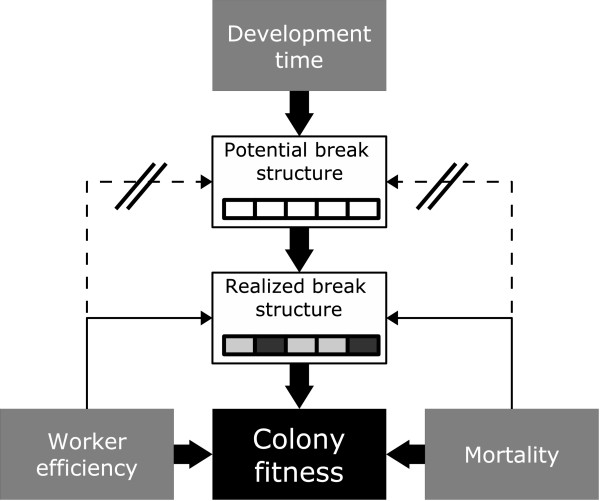
**Schematic illustration of the factors influencing the emergence of fitness increasing breaks**. Development time determines the potential temporal structure of breaks. Whether or not a potential break actually increases fitness, is determined by increase in colony survival during breaks.

The overall effect (without modifications of the mechanisms) of single time step breaks on the system performance is shown in figure 4c. It results from a superposition of figure 4a and 4b and shows the position of suitable breaks as the position of fitness peaks that surpass the critical fitness = 1 level. In this way productivity loss (4b) can be identified as the crucial process responsible for the shape of the fitness function. The mentioned mechanisms do not alter the position of the peaks even if efficiency and mortality are varied. Temporal structure is stable under a wide range of values for efficiency and mortality. Only extreme values of efficiency and mortality can cancel the break benefit completely (see fig. 5 for a schematic illustration).

**Figure 6:**
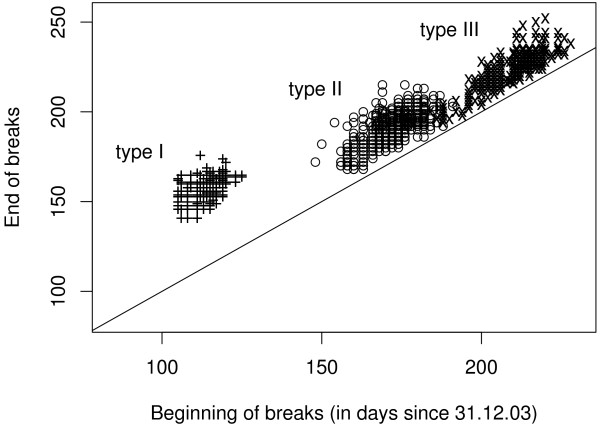
**Observed activity breaks of *L. malachurum *colonies in Wuerzburg, Germany**. Data is based on the nesting activities of 1138 nests within 13 observation patches (within an area of 4 km^2^) near Wuerzburg in northern Bavaria in 2004. Nesting activity (defined as either open nest entrance, burrowing activity, and/or the presence of a guarding bee) of each colony was recorded every other day during the whole flight season of *L. malachurum *starting in the beginning of April and lasting until the end of September (Weissel & al., in revision). Each point of the figure represents a specific combination of start and end date (Julian days) of an activity break (i.e. a sequence of days, when the nest of a single colony was closed with no signs of activity outside the nest). Both, nest closure and reopening was more or less synchronous among the nests. Symbols: + first break of a colony, ○ second break of a colony; × third break of a colony.

To assess the validity of our model we compared the break pattern with data from field observations. The activity pattern of about 1200 nests was recorded throughout the flight period in 2004. The observed pattern (fig. 6) is surprisingly similar to the pattern that was generated by the model with a particular combination of *q *and *Q*. Only the position and duration of the first break differs somewhat from the model prediction: observed breaks begin later and last longer than predicted by the model.

## Discussion

In our modelling approach the emergence of activity breaks is caused by an increased protection of developing larvae and provisioned brood cells when colonies are closed during activity breaks. The main difference from Macevicz and Oster's (1976) model is the consideration of development time. The essential predictions of our model are (1) that there are activity breaks that increase colony fitness (and might consequently be favoured by selection), (2) a clustered temporal structure of fitness increasing activity breaks similar to that observed in the field and (3) a remarkable stability of the temporal pattern within a wide range of model parameters.

As long as development time is not included in a model of colony dynamics, temporal variation in the nesting cycle can only be explained by variability in external factors: When resource availability is low (e.g. low worker efficiency rate *c*) and predation risk is high (high mortality rate *1-q*), nests should be closed to protect brood and adults of the colony, otherwise the colony should be active. Without such environmental variation the temporal course of worker numbers would always be monotonically increasing (until the optimal switching time is reached) and thus, the beneficial effect of nest closure (decreased mortality) can never outbalance the effect of productivity loss due to the wastage of time. Thus, nest closure could never increase fitness within the simple concept of temporally constant parameters [1].

However, as soon as a non negligible time for development of the larvae is taken into account [4,29], growth is delayed and the temporal course of colony size exhibits increasing as well as decreasing phases (fig. 1). With finite development time of larvae, fitness increasing activity breaks evolve as an emergent property and there is no need to assume external factors like environmental variation. Certainly environmental variation may trigger the appearance of activity breaks too, but our model provides a more general explanation that is in excellent accordance with inter- and intraspecific stability of the observed phenomenon even on a geographical scale of a species' range [31-33].

Weissel et. al (submitted, and unpublished) have shown that the temporal pattern of the colony cycle in the halictid bee *L. malachurum *depends on soil temperature, but not on resource availability or predation intensity. This result is consistent with our model prediction. We found that the potential temporal structure of active and inactive phases is only determined by development time (in relation to season length) as the main time constant of the system (fig. 5). The close relationship between development time and temperature is well known in many insect species in general [35,36] and in bees [37]. Constant mortality rates and worker efficiency just determine the occurrence of the potential breaks while temporal position of breaks is affected by the effect of soil temperature on development time. Although the influence of frequency dependent selection selection (e.g. the threat of usurpation by floater females) will tend to synchronise the temporal pattern of all externally driven activity breaks [38], the observed synchronization of colony activity in aggregations of e.g. *L. malachurum *is readily explained by the shelter a closed nest provides for the developing brood.

Activity breaks will of course reduce any mortality that is experienced by bees during foraging, e.g. by spiders, birds and wasps. In particular some crabronid wasp species of the genus *Cerceris *are specialised on hunting halictid bees as provisions for their own progeny [39]. We have observed individuals of *C. rybyensis *nesting within aggregations of *L. malachurum *so that they could easily find and paralyze workers returning from foraging trips. There are also conopid flies that wait in the nesting area and parasitize adult bees [40]. Other predators that are excluded by closing the nest are the specialized parasite bee *Sphecodes monilicornis *that violently enters nests and oviposits into brood cells [41,42] as well as predatory ants that could destroy the whole nest [40]. Notably, vespid wasps whose more or less open nests would not be much better protected by activity breaks do not show such breaks.

Although seasonal activity patterns of annual halictids with discrete broods have been described quite often [15,34,43,44], exact data on the temporal course of active and inactive phases are scarce. Weissel et al. (submitted) provide data on the seasonal activity state of about 1200 colonies of *L. malachurum *in northern Bavaria observed during a complete season in 2004 (fig. 6). For an appropriate and reasonable choice of survival rates in our model (Weissel et al., submitted) the number and temporal position of the observed activity breaks in the field are in very good accordance with our model predictions (fig. 2c). Discrepancies between field data and model predictions mainly concern the position and length of the first break. This may be due to the fact that the exact time of colony founding or beginning of egg production is difficult to determine in the field. There is also a number of simplifying assumptions in our model that particularly influence the position and length of the first break. First, we have assumed that egg production starts immediately when the colony has been founded. Second, our model does not account for any differences between founding phase and ergonomic (growth) phase of colony development. During the solitary founding phase mortality will probably be higher than later in the season, when the nest is guarded by a worker [16]. Finally, larval development time might take longer when the temperature is lower early in season. As the model assumes temporally constant mortality and development time the predicted timing of the first break can only be an imprecise estimation and a more detailed model would result in a slightly longer first activity break later in the season.

So far we did not analyse the simultaneous optimization of resource allocation and activity strategies. Although the temporal pattern of fitness increasing breaks turned out to be remarkably stable in our model there will be interactions between the optimal switching point (representing the resource allocation strategy, [1]) and break emergence (representing the resource accumulation strategy). In addition there might be constraints like egg number or egg laying rate limitation of the queens as has been observed and analysed in bumblebees [4,45-47] that have only a minor influence on activity patterns, but result in completely different optimal switching points and may even cause a different number of broods within the annual colony cycle [31].

Our model provides an explanation for the switch from continuous colony growth to reproduction with several discrete broods in social insects [14]. In contrast to the continuous growth model of Oster and Wilson [28] we provide a model for colonies which show a number of discrete broods per season, separated by distinct activity breaks. There is some evidence that the predicted transition between these two types of reproduction might occur in the field. Populations of *Halictus ligatus *have been observed exhibiting both strategies in different years [48]. The presence of adult workers within closed colonies or even worker survival for more than one brood also gives empirical indication of transitions between continuous and discrete growth [16].

Although continuous colony growth is usually taken as the standard type of colony dynamics in eusocial insects, there is no reason to assume that this is the primary state and reproduction with discrete brood periods during a season evolved from that primary state. Our model does not make any predictions about the evolutionary sequence of these two types of colony dynamics. When continuous colony growth is assumed to be the primary type of nest dynamics [28], then reproduction in discrete broods separated by activity breaks might be assumed to be a derived character. However, the contrary order seems more realistic. Hunt & Amdam (2005) analysed discrete broods as an advanced state of multivoltine reproduction of solitary species. According to their analysis social species evolved most probably from multivoltine solitary species with discrete brood events in the course of the season, as can be found in many solitary bees and wasps. Thus, the first social species most likely had discrete broods rather than continuous colony growth. On this account discrete brood reproduction can be interpreted as an evolutionary link between non-social and advanced eusocial insects like many Apidae [7]. The analysis of multivoltinism within a social context [43,49] illuminates particular aspects of the first potential transition between non-social reproduction to discrete broods while our modelling approach allows one to determine the necessary conditions for the evolution of continuous reproduction. A more detailed analysis of model parameters (season length, development time, efficiency and mortality) would be useful to determine the optimal reproductive pattern within the whole parameter space.

## Conclusion

Activity breaks are not necessarily caused by extrinsic influences. Nests are closed, whenever the resulting loss of productivity is outweighed by the benefit from increased protection of the accumulated brood. For all species and environmental conditions where the decrease in brood mortality caused by nest closure surpasses a critical level it is not necessary to assume any exogenous variability in predation or resource availability as trigger for the alternating phases of activity and inactivity. Nest cycle dynamics itself is sufficient to predict fitness increasing breaks. As productivity loss and survival increase during breaks greatly differ between species, considerable variation in activity patterns is found in annual eusocial insects ranging from species with continuous activity during the whole season to those that show extended periods of inactivity summing up to more than half the season.

## Methods

Nest dynamics was modelled as a set of coupled difference equations. The model was implemented in the programming language R version 1.7 [30]. The implementation was conducted straight forward as an iteration of the difference equations (1 and 2) with time-dependent parameters (eqns. 3, 4 and 5). Field data collection and analysis is described in the legend of figure 6.

## Authors' contributions

All authors participated in the design of the study. Oliver Mitesser developed the mathematical model, implemented and carried out the numerical calculations and drafted the manuscript. Norbert Weissel performed the field work and the presentation of field data. Erhard Strohm and Hans-Joachim Poethke conceived of the study, analysed and interpreted the numerical results and helped to draft the manuscript. All authors read and approved the final manuscript.
